# Comprehensive Rehabilitation of Post-Operative Paraplegic Patient: A Case Report

**DOI:** 10.7759/cureus.30446

**Published:** 2022-10-18

**Authors:** Dhanashree Upganlawar, Pallavi Harjpal, Snehal S Samal, Leksha Patel

**Affiliations:** 1 Physiotherapy, Ravi Nair Physiotherapy College, Datta Meghe Institute of Medical Sciences, Wardha, IND; 2 Kinesiology, Ravi Nair Physiotherapy College, Datta Meghe Institute of Medical Sciences, Wardha, IND

**Keywords:** physiotherapy, rehabilitation, wedge compression fracture, paraplegia, spinal cord injury

## Abstract

Involvement and lifestyle quality in terms of health are impacted by spinal cord injury. Spinal cord injury (SCI) patient sufferers deal with physical, social, and psychological repercussions. Annual spinal cord injuries are anticipated to range between 250,000 and 500,000. Clinical signs of SCI could include a partial or complete sensation loss and/or motor activity below the site of the damage. While quadriplegia could develop from injuries to the cervical region, paraplegia could result from injuries to the lower thorax. The most popular technique for predicting outcomes after the SCI is the International Standards for Neurological Classification of Spinal Cord Injury (ISNCSCI), which was created in partnership with the American Spinal Injury Association. A 28-year-old patient visited our hospital with complaints of reduced strength in both lower limbs and unable to walk. For those with whole or incomplete paraplegia, regaining independent mobility during the chronic phase is the most crucial goal. Bed mobility training, upper limb strengthening, trunk control, and intervention were started. SCI is an example of a low-incidence ailment that does not generate sufficient market demand to sustain the development of specialist services in distant places. The rehabilitation strategy should include weight-bearing mat exercises, home exercise programs, and ambulation orthoses. Early physiotherapy participation on the side of the patient allowed him to avoid major secondary issues including bed sores and joint contractures. One of the crucial components of the recovery process for those with spinal cord injuries is physical therapy.

## Introduction

Spinal cord injury (SCI) is a challenging and quickly developing condition. Participation and health-related quality of life (HR-QOL) are impacted by SCI. Spinal cord injury patients are affected physically, socially, and psychologically [1]. A strategic plan aids in determining a person's requirements, priorities, and expectations for restoration. Annual spinal cord injuries are anticipated to range between 250,000 and 500,000 [2]. More than 90% of SCI instances have traumatizing etiologies that include incidents like automobile accidents, assaults, sports, or falls. Clinical indications of SCI may include a partial or complete loss of perception and/or functional ability immediately below the level of injury, depending on the extent and location of the damage [3]. Injuries in the lower thorax can result in paraplegia, whereas those in the cervical region might result in quadriplegia [4].

There are two stages in SCI: the first one, which consists of two mechanical injuries, glial cells and neurons and their surroundings; and the second one, which includes vasculature, the environmental deterioration that is pervasive in spinal cord cells [4]. Fractures and vertebral dislocation are common outcomes of acute SCI, which frequently stems from rapid trauma to the spine [5]. The approach is most frequently used to predict outcomes after SCI. The American Spinal Injury Association (ASIA) and the International Standards for Neurological Classification of Spinal Cord Injury (ISNCSCI) together developed the disability scale [6]. Around 5% and 10% of polytrauma patients experience spinal fractures or dislocations, with the lumbar or dorsal spine accounting for 65%-80% of these injuries [7]. Paraplegia, or the paralysis of the entire trunk and both lower extremities, results from damage to the dorso-lumbar region of the spinal column. The lower limb and bowel and bladder may be compromised based on the extent of the impairment, whereas the arm may continue to function correctly [8].

Depending on the kind of injury, there may or may not be bowel and bladder involvement, in which case they have neurogenic bowel and bladder that are spastic or flaccid [6]. A patient's standard of living may suffer as a result of having restricted and altered mobility, the ability to care for oneself, and the ability to engage in beloved social activities if muscles below the level of injury are paralyzed. Patients with paraplegia can gain improvement if physical therapy interventions are started earlier as possible. Through target, practice, and repetition, initial activities training in rehabilitation can enhance performance [9]. In such patients, our main target is focusing on bed mobility training, transferring activities, and making the patient skillful to execute his activities of daily living (ADLs). A task is allotted to the patient, and we teach them how to perform it within a pain-free limit [8].

## Case presentation

Patient information

A 28-year-old male citizen of Gadchiroli had a road traffic accident one year ago. He was taken to a local hospital for treatment. He was diagnosed with wedge compression fracture of the D6 vertebra and was advised for operation. He was taken to Nagpur for an operation. Now he visited our hospital with the complaint of bilateral lower limb weakness, absence of bowel and bladder control, and unable to walk. He was advised for physiotherapy on September 7, 2022. The patient’s blood pressure was 130/80 mmHg, pulse rate was 82 b/min, and respiratory rate was 13 breaths/min on physical examination.

Clinical findings

The patient’s build was mesomorphic, with an attitude of limb: ankle in slight plantar flexion and hip externally rotated. During a neurological examination, sensations below the D6 level were absent. In both lower limbs, the tone was spastic grade 1 according to the Modified Ashworth Scale (MAS). All the deep tendon reflexes were present. Both upper limbs were neurologically normal. The patient underwent intermittent catheterization because the bowel and bladder were involved. Magnetic resonance imaging (MRI) finding reveals fixation of screw seen in D4-D8 vertebra and compression fracture of D6 with no retropulsion of vertebral body. An X-ray of the dorso-lumbar spine's anteroposterior (AP) and lateral views indicates spinal fixation at levels D4 and D8, as well as a compression fracture at level D6.

Investigations

**Figure 1:**
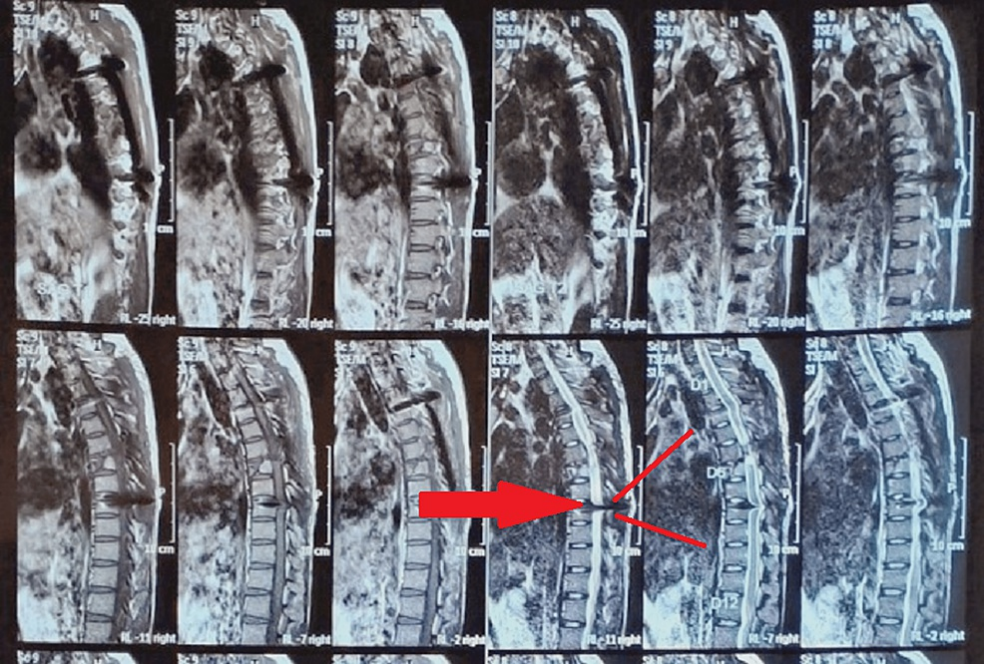
Magnetic resonance image of the patient The red arrow and lines indicate the level of injury.

Magnetic resonance imaging (MRI) was done, displayed in Figure 1.

Physiotherapy interventions

**Figure 2:**
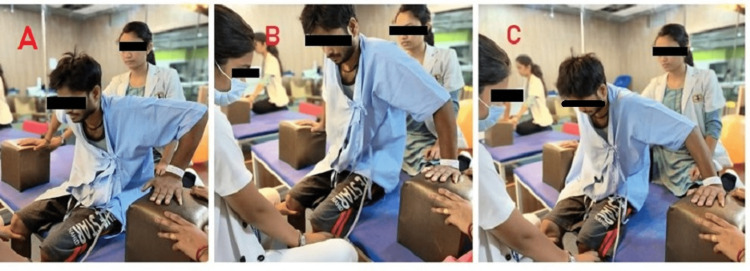
Patient performing scooting for weight shifting A: The initial position for scooting. B: The patient shifts the weight and tries to stand. C: The patient is shifted to another side.

**Figure 3:**
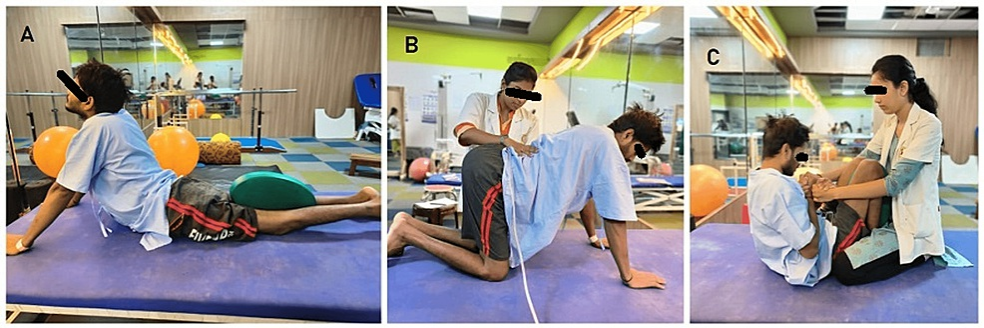
Patient performing physiotherapy intervention A: The patient is in a cobra pose. B: The patient is quadruped position. C: The patient is doing crunches.

Management for this patient was primarily focused on preventing secondary complications such as bed sores, deep vein thrombosis, and respiratory complications. Bed mobility training was started. Strengthening of both upper limbs was initiated with a weight cuff of 1 kg. For lower limb stretching exercises, positioning, bed mobility exercises, trunk stability exercises, exercises to improve static and dynamic balance, scooting, pelvic tilt, and weight shift exercises were given. Table 1 indicates the physiotherapy rehabilitation protocol (Figures 2A-2C, 3A-3C).

**Table 1:** Physiotherapy rehabilitation protocol

Problems faced by the patient	Goals to improve problems	Physiotherapy intervention
1. Secondary complications such as bed sores, deep vein thrombosis, etc.	To prevent this secondary complication.	Ankle-toe movement every two hours and changing the position.
2. Respiratory complications	To prevent breathing difficulties.	Techniques for breathing involve pursed lip breathing, diaphragmatic breathing, and thoracic expansion.
3. Tightness (tendon Achilles (TA), hamstring)	To reduce the tightness.	Stretching of TA, stretching of the hamstring (15-sec hold × 3 reps).
4. Mobility issue	To improve mobility.	Bed rolling, passive movements for bilateral lower limbs. Back extension exercise (Figure 3A).
5. Bowel and bladder	To strengthen the pelvic muscles.	Kegel’s exercises, transverse abdominal contraction, hip abductor, and adductor roll.
6. Core muscle weakness	To strengthen core musculature.	Crunches both straight and diagonally (5 sec hold × 10 reps: 1 set) of each (Figure 3C). Push-ups on arms (10 reps × 1 set).
7. Trunk muscle weakness	To strengthen the trunk muscles and improve stability.	In the quadruped position shifting of weight side to side, forward, and backward (Figure 3B) (10 reps × 1 set).
8. Balance	To improve static and dynamic balance.	Scooting (Figure 2A-2C), weight shifts in sitting position side to side, forward, and backward (10 reps × 1 set), maintain static balance (20 sec hold with eyes closed and open × 3 reps).
9. Difficulty in maintaining a standing position	To make the patient stand.	Supported standing.
10. Sensations were absent	Sensory reeducation.	Using different textures such as feathers, cotton cloth, Turkish cloth, rubbing sand, silk cloth, etc., from distal to proximal.
11. Disuse may lead to reduce in upper extremity strength	Maintenance of upper extremity strength.	Upper limb strengthening with 1 kg of weight cuff (10 reps × 2 sets).

Outcome measures

**Figure 4:**
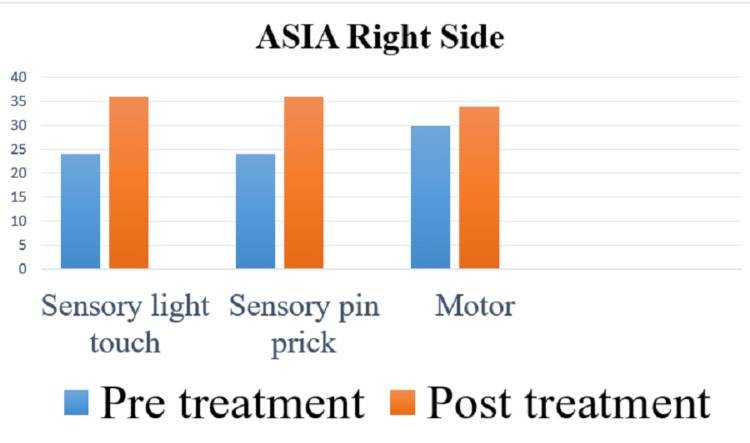
Pre- and post-treatment score of the right side on ASIA ASIA: American Spinal Injury Association.

**Figure 5:**
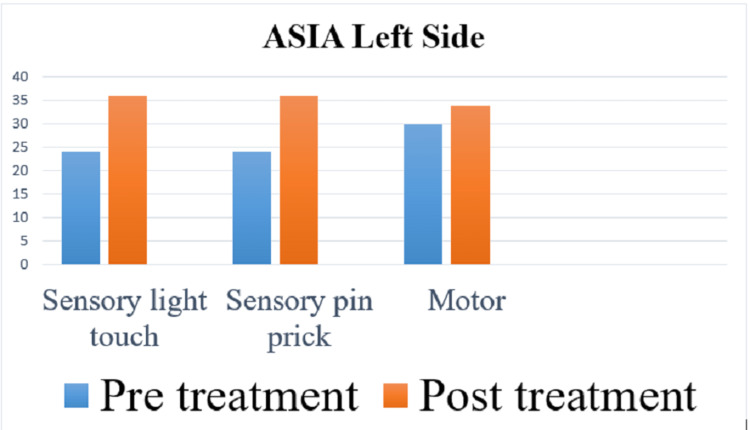
Pre- and post-treatment score of the left side on ASIA ASIA: American Spinal Injury Association.

**Figure 6:**
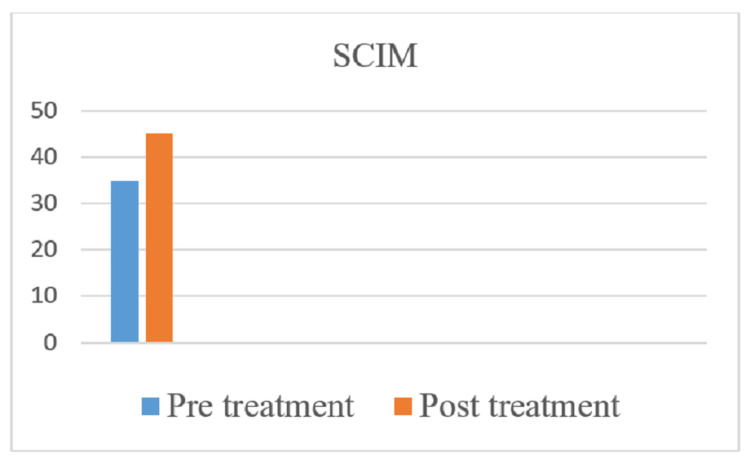
SCIM score improvement post-rehabilitation SCIM: Spinal Cord Independence Measure.

**Figure 7:**
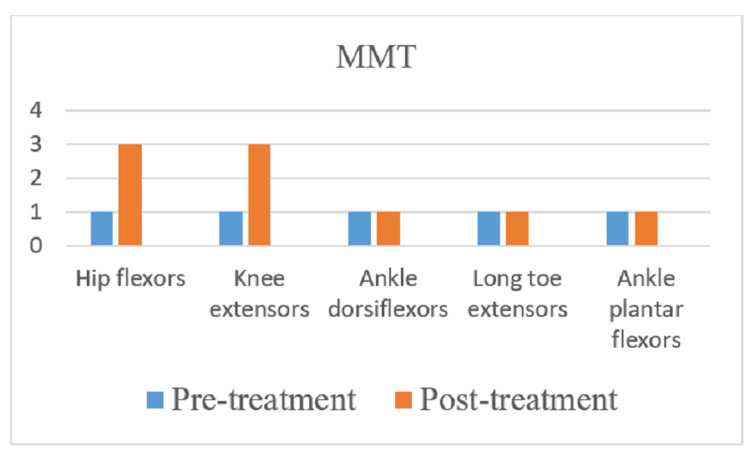
Improvement in lower limb MMT post-rehabilitation MMT: Manual Muscle Testing.

The American Spinal Injury Association (ASIA) scale was used to measure the outcome of rehabilitation. In the pre-treatment score for the right side, the sensory was 48 and the motor score was 30, and for the left side, the sensory was 48 and the motor was 30, and the overall neurological level was T5. In the post-treatment score for the right side, the sensory is 72 and the motor is 34, and for the left side, the sensory is 72 and the motor is 34, and the overall neurological level is T7. Spinal Cord Independence Measure (SCIM) and Manual Muscle Testing (MMT) were used as outcome measures. Figures 4-7 indicate the outcomes of the intervention.

## Discussion

This case study intends to demonstrate how effective surgical and physical therapy treatment can be provided to a patient who has suffered a serious spinal cord injury. During the chronic phase, achieving independent mobility is the most important objective for people with total and incomplete paraplegia [10]. Patients with chronic SCI require a long-term, intense rehabilitation program. Reducing the rate of death due to complications requires aggressive prevention and control of any problems that may arise [10].

Weight-bearing mat exercises, home exercise routines, and ambulation orthoses should all be part of the rehabilitation protocol [11]. Every exercise was performed three times daily. After the rehabilitation program, he was able to restart his ADL on his own [12]. The first coordinated effort is to determine critical markers of high-quality rehabilitation for people with SCI that may be integrated into routine clinical care. SCI is an example of a low-incidence ailment that does not generate enough of a market to sustain the establishment of specialist services in distant locations [13].

However, the designs and the functions must be enhanced so that they help users to achieve their goals more effectively with mechanical orthotic devices. To increase physical fitness and survival in these patients, adequate respiratory treatment for pulmonary function optimization may be essential. However, respiratory care is generally used in physical rehabilitation therapy [14].

In this case, when a patient came for physiotherapy on the first day, he was unable to move both lower limbs. As the patient came one year later to take the physiotherapy treatment, it was difficult to gain recovery as early as possible. But as we started giving the treatment, our main focus was on bed mobility activities, transferring activities, and making the patient perform his ADLs [8]. After two months of treatment, he was able to initiate the movement and he has achieved unsupported sitting. If early physiotherapy is not adequately implemented, there is a potential that recovery time will take longer. Sometimes it might result in the emergence of strange synergy patterns [9].

## Conclusions

This case study's findings show the importance of effective physical therapy interventions and exercises in enabling patients to carry out activities of daily living. The patient's early participation in physical therapy helped him to prevent serious secondary problems such as pressure sores and joint contractures. One of the crucial aspects of spinal cord injury patients' recovery is early physical therapy intervention. This study also suggests that if intervention is followed five days per week three times a day, it will be helpful for patients to gain recovery earlier.
